# Neurally Adjusted Ventilatory Assist (NAVA) or Pressure Support Ventilation (PSV) during spontaneous breathing trials in critically ill patients: a crossover trial

**DOI:** 10.1186/s12890-017-0484-5

**Published:** 2017-11-07

**Authors:** Juliana C. Ferreira, Fabia Diniz-Silva, Henrique T. Moriya, Adriano M. Alencar, Marcelo B. P. Amato, Carlos R. R. Carvalho

**Affiliations:** 1 Divisao de Pneumologia, Instituto do Coracao, Hospital das Clinicas HCFMUSP, Faculdade de Medicina, Universidade de Sao Paulo, São Paulo, SP Brazil; 2 Biomedical Engineering Laboratory, Escola Politécnica da Universidade de Sao Paulo, São Paulo, SP Brazil; 3 Instituto de Física, Universidade de São Paulo, São Paulo, SP Brazil

**Keywords:** Continuous positive airway pressure, Positive-pressure respiration, Respiration, artificial, Ventilator weaning

## Abstract

**Background:**

Neurally Adjusted Ventilatory Assist (NAVA) is a proportional ventilatory mode that uses the electrical activity of the diaphragm (EAdi) to offer ventilatory assistance in proportion to patient effort. NAVA has been increasingly used for critically ill patients, but it has not been evaluated during spontaneous breathing trials (SBT). We designed a pilot trial to assess the feasibility of using NAVA during SBTs, and to compare the breathing pattern and patient-ventilator asynchrony of NAVA with Pressure Support (PSV) during SBTs.

**Methods:**

We conducted a crossover trial in the ICU of a university hospital in Brazil and included mechanically ventilated patients considered ready to undergo an SBT on the day of the study. Patients underwent two SBTs in randomized order: 30 min in PSV of 5 cmH_2_O or NAVA titrated to generate equivalent peak airway pressure (Paw), with a positive end-expiratory pressure of 5 cmH_2_O. The ICU team, blinded to ventilatory mode, evaluated whether patients passed each SBT. We captured flow, Paw and electrical activity of the diaphragm (EAdi) from the ventilator and used it to calculate respiratory rate (RR), tidal volume (VT), and EAdi. Detection of asynchrony events used waveform analysis and we calculated the asynchrony index as the number of asynchrony events divided by the number of neural cycles.

**Results:**

We included 20 patients in the study. All patients passed the SBT in PSV, and three failed the SBT in NAVA. Five patients were reintubated and the extubation failure rate was 25% (95% CI 9–49%). Respiratory parameters were similar in the two modes: VT = 6.1 (5.5–6.5) mL/Kg in NAVA vs. 5.5 (4.8–6.1) mL/Kg in PSV (*p* = 0.076) and RR = 27 (17–30) rpm in NAVA vs. 26 (20–30) rpm in PSV, *p* = 0.55. NAVA reduced AI, with a median of 11.5% (4.2–19.7) compared to 24.3% (6.3–34.3) in PSV (*p* = 0.033).

**Conclusions:**

NAVA reduces patient-ventilator asynchrony index and generates a respiratory pattern similar to PSV during SBTs. Patients considered ready for mechanical ventilation liberation may be submitted to an SBT in NAVA using the same objective criteria used for SBTs in PSV.

**Trial registration:**

ClinicalTrials.gov (NCT01337271), registered April 12, 2011.

**Electronic supplementary material:**

The online version of this article (10.1186/s12890-017-0484-5) contains supplementary material, which is available to authorized users.

## Background

Mechanical ventilation is a core component of life support for critically ill patients that is applied to approximately one third of patients admitted to the Intensive Care Unit (ICU) [1]. However, there are several complications associated with mechanical ventilation and it should be discontinued as soon as clinical condition improves [2, 3]. Transition from controlled mechanical ventilation to unassisted breathing, known as liberation from mechanical ventilation, should be as rapid as possible, rather than a gradual process of decreasing ventilatory support [3, 4]. The process of liberation usually involves the application of spontaneous modes of ventilation like Pressure Support Ventilation (PSV), and more importantly, daily assessment of readiness to undergo a spontaneous breathing trial (SBT) [5, 6].

Typically, an SBT consists of removing or minimizing ventilatory support for 30–120 min and observing if the patient tolerates the challenge. This can be accomplished either using a t-tube or with low levels of ventilator support, using continuous positive airway pressure (CPAP) or 5–7 cmH_2_O of pressure support. However, there is no consensus as to what is the best method to perform the SBT [7, 8].

Neurally adjusted ventilatory assist (NAVA) is a new mode of mechanical ventilation that uses the electrical activity of the diaphragm (EAdi) to trigger and cycle inspiratory assistance and provide it in proportion to the patient’s effort [9–12]. Studies showed that NAVA improves patient-ventilator synchrony and reduces the risk of over-assistance [13–19], which makes it an attractive alternative for patients experiencing clinically significant asynchrony on PSV. However, there are no studies describing the breathing pattern and performance of NAVA during SBTs. Under current treatment paradigms, when deemed ready to undergo an SBT, patients ventilated with NAVA need to perform the SBT either in a T-tube, which provides less objective monitoring of respiratory parameters, or in PSV, which may increase the occurrence of patient-ventilator asynchrony [20–22]. Therefore, we designed a pilot trial to assess the feasibility of using NAVA during SBTs, to estimate the rate of success in the SBT on NAVA and to compare the breathing pattern and patient-ventilator asynchrony of NAVA with PSV during SBTs. We hypothesized that NAVA would decrease patient-ventilator asynchrony compared to PSV.

## Methods

### Study population

We conducted a crossover trial in the respiratory ICU of a university hospital in São Paulo, Brazil, from June 2011 to September 2013. The institution’s ethics committee, CAPpesq (University of Sao Paulo Medical School Ethics’ committee) approved the study (ID 0336/10) and a family member of each participant provided informed consent before inclusion. The study was registered at ClinicalTrials.gov (NCT01337271).

We included a convenience sample of 20 patients since data on the performance of NAVA during SBTs was not available in the literature. We prospectively enrolled consecutive patients admitted to the ICU during the study period who had received mechanical ventilation for more than 48 h and who the ICU team considered to be ready to undergo an SBT. Patients were included only if this was the first SBT attempt. Exclusion criteria were age less than 18 years, pregnancy, tracheotomy, participation in other clinical trials, and contraindications to the placement of the esophageal catheter (nasal pathologies, facial trauma or burns, or esophageal varicose or gastro esophageal bleeding in the past 30 days).

### Study protocol

Patients were ventilated with the Servoi Ventilator (Maquet, Sweden), using heated humidification. We left the ventilator settings at baseline to the discretion of the ICU team.

As previously described [23, 24], we measured EAdi using a dedicated NAVA catheter (Maquet, Sweden), which has a multiple array of electrodes placed at its distal end to capture diaphragmatic electrical activity. A specific function of the ventilator was used to guide the correct positioning of the catheter.

Before initiating the SBTs, we set PSV to 5 cmH_2_O and positive end-expiratory pressure (PEEP) to 5 cmH_2_O for 5 min and we titrated the NAVA level to generate a peak inspiratory pressure of 10 cmH_2_O [14].

All patients underwent two SBTs, one in PSV, and one in NAVA, separated by a washout period of 1 h, during which they received ventilation with baseline settings. We randomized the order of the SBTs using a computer generated randomization list, concealed by sequentially numbered, opaque, and sealed envelopes.

SBTs in PSV used a pressure support of 5 cmH_2_0 and PEEP of 5 cmH_2_O. We did not change the baseline flow-trigger sensitivity, cycling-off criteria, or FIO_2_. SBTs in NAVA used the NAVA level titrated as described above, a PEEP of 5 cmH_2_O, and baseline FIO_2_ and flow-trigger sensitivity. NAVA triggering sensitivity and cycling off are fixed at 0.5 μV and 70% of EAdi peak, respectively. NAVA could be triggered either by EAdi or flow triggering, whichever happened first.

The SBTs lasted 30 min each. We suctioned the endotracheal tube before the beginning of each test and made no changes in ventilator setting during the SBTs. The ICU team was responsible for evaluating the patient during the SBT and deciding whether or not the patient had passed each test. We blinded the ICU team to the order of the SBTs to prevent bias on the SBT evaluation. We partially covered the ventilator screen during the SBTs to hide the ventilator waveforms in order to prevent unbinding. Respiratory rate, tidal volume, and minute volume were visible on the right side of the screen. We instructed clinicians caring for the patient to interrupt the test according to the standard of care criteria for SBT failure: respiratory rate greater than 35 rpm or less than 6 rpm; hypoxemia; changes in mental status; new onset of cardiac arrhythmia, tachycardia, or bradycardia; diaphoresis; or signs of respiratory distress, but investigators did not interfere with clinicians decision to interrupt the test in any circumstance . If the test was not interrupted for one of these objective criteria, investigators asked ICU clinicians if the patient had failed or passed the SBT at the end of the SBT. If the patient failed the first SBT, the ICU team was instructed to allow the second SBT after the washout period unless they considered it to be unsafe for the patient. The investigators did not participate in the decision to interrupt an SBT or to extubate the patient at the end of the second SBT. At the end of the protocol, we disclosed the order of the SBTs to the ICU team and instructed them to use the result of the SBT in PSV to decide whether or not to extubate the patient. We followed patients until ICU discharge and defined extubation failure as the need for reintubation in the first 48 h after extubation.

### Data acquisition and analysis

We performed blood gas analysis at baseline, at the end of the washout period, and at the end of each SBT. Dedicated software (NAVA Tracker V.4.0; Maquet, Sweden) acquired the airway pressure, flow, and EAdi from the ventilator at a sampling rate of 100 Hz for periods of 3 min at each of the following times: at baseline, during the last 10 min of the washout period, and every 10 min during each SBT. We processed and analyzed the data using MatLab (Mathworks, MA, USA), which automatically detected the initiation and termination of inspiratory efforts and ventilator cycles, and calculated peak airway pressure (Paw), neural inspiratory time (TIneural), ventilator inspiratory time (TIvent), respiratory rate (RR), tidal volume (V_T_) in mL/kg of ideal body weight, and ΔEAdi, which is defined as EAdi peak minus EAdi minimal. Neuro ventilatory efficiency was calculated as tidal volume (in mL) divided by ΔEAdi, in μV [25]. We excluded cycles with artifacts and, for every subject, we averaged all the cycles in each three-minute recording to generate mean values for the above variables.

Detection of major asynchrony events employed both visual inspection and processing of the recordings of Paw, flow, and EAdi waveforms. We define these events below.

- Auto triggering: the ventilator cycle was not preceded by an inspiratory effort.
- Triggering delay: the delay between the start of patient effort and triggering was >25% of the mean inspiratory time.
- Ineffective effort: an inspiratory effort not accompanied by a ventilator cycle.
- Double triggering: two cycles separated by an expiratory time that was less than half of the mean inspiratory time.
- Cycling delay: inspiratory time greater than twice the mean inspiratory time.
- Premature cycling: inspiratory time less than half the mean inspiratory time.

As previously described [26, 27], we calculated the asynchrony index (AI) as the number of cycles with asynchrony divided by the number of monitored neural cycles, expressed as a percentage.

### Statistical analysis

Statistical analysis used Stata 12.0 (Stata, TX, USA). The analyses express continuous variables by their median and the 25–75% interquartile range (IQR). The McNemar test was used to compare the success rates of SBTs in NAVA and PSV. The paired Wilcoxon signed-rank test was used to compare continuous variables during the SBT in NAVA and PSV. Unpaired t tests and chi-square tests were used to compare patients who had successful extubation with those with extubation failure. We considered a *p* value less than 0.05 to be significant.

## Results

The trial included 20 patients who completed the protocol. Median age was 60 years old, ranging from 19 to 82 years old; seven patients were female and 13 were male. Table 1 shows their baseline characteristics and ventilator settings on the day of inclusion in the study. All patients were being ventilated in PSV at the time of inclusion. The most common causes of respiratory failure were chronic obstructive pulmonary disease (COPD) exacerbation and pneumonia and the median duration of mechanical ventilation was 6 days. Ventilatory parameters at baseline are shown in the (Additional file 1: Table S1).

**Table 1:** Patient’s demographics and ventilator settings at baseline

ID	SAPS 3	Cause of Resp. Failure	Duration of MV (days)	PSV (cmH_2_O)	PEEP (cmH_2_O)	Cycling off	FIO_2_
1	67	COPD exacerbation	5	10	5	30%	0.3
2	71	COPD exacerbation	2	17	7	35%	0.4
3	70	COPD exacerbation	7	12	6	40%	0.35
4	84	Trauma	12	12	5	35%	0.3
5	95	Sepsis	6	7	8	20%	0.35
6	97	Cardiac arrest	3	10	8	30%	0.3
7	55	Coma	9	9	8	30%	0.4
8	65	Metabolic acidosis	8	8	6	40%	0.35
9	71	Pneumonia	3	6	6	30%	0.3
10	39	Pneumonia	5	7	6	25%	0.25
11	76	Pneumonia	7	8	8	30%	0.35
12	48	Drowning	7	10	10	15%	0.3
13	76	Pleural effusion	7	10	6	30%	0.3
14	57	Cardiac failure	10	10	8	30%	0.35
15	50	COPD exacerbation	4	8	5	60%	0.35
16	65	Pneumonia	3	6	8	25%	0.35
17	69	Pneumonia	6	10	5	25%	0.3
18	44	COPD exacerbation	11	12	6	40%	0.5
19	58	COPD exacerbation	5	15	8	50%	0.5
20	32	COPD exacerbation	4	10	5	30%	0.35

*SAPS 3* Simplified acute physiology score 3, *Resp. Failure* respiratory failure, *MV* Mechanical ventilation, *PSV* Pressure Support Ventilation level on the day of the study, *PEEP* positive end-expiratory pressure on the day of the study, *FIO*_*2*_ inspired fraction of oxygen, *COPD* Chronic Obstructive Pulmonary Disease. Duration of mechanical ventilation is shown in days before inclusion in the trial

**Fig. 1:**
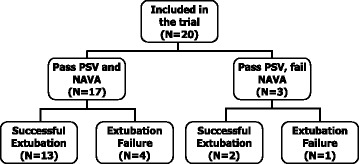
Outcomes of the Spontaneous Breathing Trials (SBT) in Pressure Support Ventilation (PSV) and Neurally Adjusted Ventilatory Assist (NAVA) and extubation outcomes for the patients included in the trial

All patients underwent the two SBTs. NAVA was well tolerated and none of the SBTs were interrupted by the ICU team. The mean NAVA level used was 0.7 cmH_2_O/μV, ranging from 0.2 to 1.4 cmH_2_O/μV. Figure 1 shows the results of the SBTs and the outcomes of extubation for all patients. Individual ventilatory parameters during the SBTs are shown in the (Additional file 2: Table S2). All patients passed the SBT in PSV, but three failed the SBT in NAVA. The SBT success rate was 85% (95% CI 62–97%) in NAVA and 100% (95% CI 83–100%) in PSV (*p* = 0.250). Five patients were reintubated and the extubation failure rate was 25% (95% CI 9–49%).

Patients who were reintubated had higher SAPS 3 compared to among those who were not reintubated (78 ± 12 vs. 59 ± 17, *p* = 0.039). They also had more trigger delay at baseline: 33% (22% - 46%) of the cycles compared to 5% (3%–14%) for those who were not reintubated, *p* = 0.015. Duration of mechanical ventilation prior to the first SBT and a diagnosis of COPD were not associated with a higher risk of reintubation.

Median respiratory rate during the SBTs was 27 rpm (17–30) in NAVA and 26 rpm (20–30) in PSV (*p* = 0.559), and tidal volume, in mL/Kg of ideal body weight, was 6.1 (5.5–6.5) in NAVA and 5.5 (4.8–6.1) in PSV (*p* = 0.076). The median ΔEAdi was comparable for NAVA and PSV, while peak Paw was greater in NAVA than in PSV (Table 2). Blood gases were within normal range and did not change significantly from baseline (Table 2).

**Table 2:** Respiratory parameters during the Spontaneous breathing trials

Variable	NAVA (*n* = 20)	PSV (*n* = 20)	*p* value
RR (rpm)	27 (17–30)	26 (20–30)	0.560
Vt/Kg (mL/Kg)	6.1 (5.5–6.5)	5.5 (4.8–6.1)	0.076
ΔEAdi (μV)	10.3 (5.2–22.9)	10.2 (6.4–20.6)	0.376
TIvent (s)	0.81 (0.66–1.11)	0.76 (0.68–0.91)	0.007
NVE (mL/μV)	34.5 (23.3–66.8)	35.4 (19.9–46.8)	0.920
Paw (cmH_2_O)	14.0 (12.9–15.2)	11.2 (11–11.7)	<0.001
pH	7.46(7.42–7.49)	7.46 (7.40–7.49)	0.947
pCO2 (mmHg)	40 (35–43)	40 (36–45)	0.188

Values are medians (25%–75% interquartile range). *RR* respiratory rate, *V*_*T*_ */kg* tidal volume per kilogram of predicted body weight, *ΔEAdi* Delta Electrical activity of the diaphragm, *TIvent* ventilator inspiratory time, *NVE* neuroventilatory efficiency, calculated as tidal volume divided by ΔEAdi, *Paw* airway pressure. pH and pCO_2_ from blood gas analysis. The *p* values were obtained with the Wilcoxon sumrank test

When compared with patients who passed their SBT in NAVA, the three patients who failed the SBT in NAVA had shorter TIvent in NAVA (0.53 ± 0.11 s, vs. 0.92 ± 0.21 s, *p* = 0.008). There were non-significant trends towards higher RR in patients who failed the SBT in NAVA (31 ± 5 rpm vs. 24 ± 7 rpm, *p* = 0.136) and lower neuro ventilatory efficiency (64 ± 24 mL/μV vs. 35 ± 22 mL/μV, *p* = 0.06). One of the patients was in delirium and became more agitated during his SBT in NAVA.

**Fig. 2:**
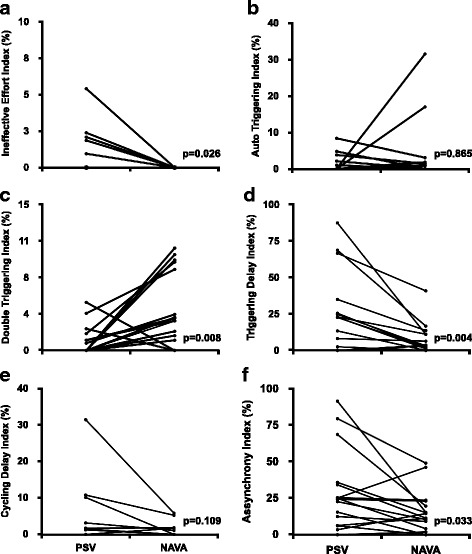
Prevalence of each type of asynchrony in Pressure Support Ventilation (PSV) and Neurally Adjusted Ventilatory Assist (NAVA) for each patient as well as the total asynchrony index. **a** Ineffective effort index; **b** Auto triggering index; **c** Double triggering index; **d** Triggering delay index; **e** Cycling delay index; **f** Asynchrony index

**Fig. 3:**
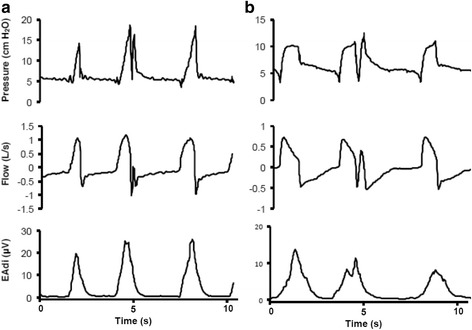
Tracings for Airway Pressure, Flow, and Electric Activity of the Diaphragm (EAdi) showing double-triggering in Neurally Adjusted Ventilatory Assist (NAVA) and Pressure Support Ventilation (PSV). **a** double triggering in NAVA; notice that despite the elevation of airway pressure on the second breath during the double triggering event, no flow is delivered to the patient, therefore no extra tidal volume was delivered in this second breath. **b** double triggering in PSV; the second breath during the double triggering event elevated the airway pressure and caused a positive inspiratory flow, therefore delivering a second tidal volume

Of the 20 patients, three had excessive oscillation on EAdi waveform recordings that allowed us to calculate breathing parameters but prevented accurate estimation of the beginning of patient effort, which is essential to detect triggering asynchrony. Therefore, these three patients were excluded from the asynchrony analysis. Of the remaining 17 patients, five patients displayed ineffective efforts during PSV, and NAVA abolished this type of asynchrony (Fig. 2a, *p* = 0.026). Auto triggering was comparable between NAVA and PSV (Fig. 2b), (*p* = 0.865). Double triggering was more common in NAVA than PSV (Fig. 2c, *p* = 0.008), but the second breath usually had zero flow in NAVA, while in PSV the flow was usually positive (Fig. 3). Triggering delay (Fig. 2d) was more common in PSV than in NAVA (*p* = 0.004) and cycling delay (Fig. 2e) was similar in both modes (*p* = 0.109). Premature cycling was rare and similar for both modes (*p* = 0.614). AI was greater than 10% in 12 patients in PSV and 11 patients in NAVA. The asynchrony index had a median of 24.3% (6.3–34.3) in PSV and 11.5% (4.2–19.7) in NAVA (*p* = 0.033).

## Discussion

In this crossover pilot trial including intubated patients considered ready for mechanical ventilation liberation, we found that an SBT in NAVA was usually well tolerated, had similar respiratory rate and tidal volumes to an SBT in PSV, and overall similar clinical performance. We also found that major asynchrony events were common during the SBTs and that most patients had an asynchrony index greater than 10%. NAVA significantly reduced the asynchrony index by reducing triggering delay and cycling delay, but caused more double triggering.

To our knowledge, this the first study describing the performance of NAVA during an SBT. While previous studies have evaluated this new mode for patients under mechanical ventilation during the transition from controlled ventilation to spontaneous breathing [13–16], none of them used NAVA continuously until extubation. In the largest multicentric clinical study using NAVA, Demoule et al. randomized 128 patients to be ventilated with NAVA or PSV for up to 14 days, but the spontaneous breathing trials were performed with PSV (or in a T-tube in a few cases), not NAVA [28]. EAdi monitoring during SBTs in PSV or T-tube has also been used to predict weaning success [29, 30] and to monitor patient effort after extubation in a high risk patient [31]. Previous studies had shown lower tidal volumes and greater respiratory rates in NAVA compared to PSV, and such a pattern could interfere with the interpretation of an SBT. Without data on how NAVA performs during an SBT, previous studies that used NAVA during the weaning phase performed their daily SBTs either with a T-tube, PSV or CPAP [20–22, 32]. The T-tube has been shown to yield similar results compared to minimal support in PSV during SBTs [33], but it doesn’t allow for precise FIO_2_ setting nor does it provide breath-by-breath respiratory parameters. Using PSV allows for closer monitoring, but for patients who are being ventilated with NAVA because of asynchronies in PSV, using PSV for the SBT could decrease the rate of SBT success and delay extubation.

### Breathing pattern

We did not observe lower tidal volumes or higher respiratory rates in NAVA compared to PSV. This may be because these changes were described in studies in which PSV levels were high and over-assistance was common [13–19, 34], while in our study, patients were receiving minimal support in both PSV and NAVA. We observed greater Paw in NAVA than in PSV (Table 2) despite having titrated NAVA before randomization to generate equivalent Paw in NAVA and PSV. But because Paw in NAVA is proportional to patient effort, greater Paw during the SBT in NAVA occurred because ΔEAdi was greater during the SBTs (both NAVA and PSV) than during the titration period. However, this difference is unlikely to have resulted in more assistance during the SBTs in NAVA compared to SBTs in PSV, because ΔEAdi, RR and V_T_ were similar in NAVA and SBT; moreover, if ventilatory support during the SBT in NAVA had been considerably greater than during the SBT in PSV, we would expect lower rates of SBT failure in NAVA, which was not the case.

These results suggest that patients being ventilated with NAVA during the weaning period may be screened for extubation readiness with NAVA using the same objective criteria for SBT failure used for SBTs in PSV or a T-tube.

### Asynchrony

Asynchrony events were common among this population and were, for the most part, overlooked by the clinicians caring for the patients [35]. The AI was greater than 10%, which is considered clinically significant, in 12 (71%) patients in PSV and 11 (64%) patients in NAVA. Compared to PSV, NAVA decreased total AI, which agrees with most previous studies comparing NAVA and PSV [14–16, 19, 36]. What is novel in our results is that the AI was lower in NAVA even in the absence of over-assistance with PSV. In previous studies, NAVA decreased the total asynchrony index mostly by decreasing ineffective efforts [13–16, 36]. In our study, ineffective efforts were uncommon because over-assistance, its most important risk factor, is not expected during SBTs. Only five patients showed ineffective efforts during PSV, which were abolished with NAVA.

Triggering delay was the most prevalent type of asynchrony and NAVA significantly reduced its occurrence compared to PSV. Few previous authors have included triggering delay into the computation of total AI [37] because its detection requires direct monitoring of patient inspiratory effort. Since triggering delay may cause considerable discomfort [38] and is easily measured with the NAVA catheter [36, 39, 40], we added it to our computation of total AI. Therefore, comparisons of our results to previous studies that did not include this type of asynchrony should take this difference into account.

NAVA had no significant impact in cycling asynchrony in comparison with PSV. Some previous studies showed a reduction in cycling delay with NAVA [13–16] but we observed an overall low incidence of cycling delay, probably because inspiratory assistance during the SBTs was minimal. Double triggering was more common in NAVA compared to PSV, which agrees with previous results [13, 19]. Therefore, clinicians caring for patients ventilated with NAVA should pay special attention to this type of asynchrony and take measures to minimize it. Interestingly, double triggering in NAVA did not result in breath staking and high tidal volumes, which is a concern related to double triggering in assisted-controlled modes [41], because the second breath usually resulted in zero flow, as shown in Fig. 3. No flow was present on the second breath because, during inspiration, NAVA delivers inspiratory flow to generate a target airway pressure equal to EAdi times the NAVA level. When double triggering occurs, if the tidal volume delivered in the first breath has not been completely exhaled, airway pressure at the beginning of the second breath may be greater than the target airway pressure calculated by NAVA, and thus the ventilator does not deliver more flow.

### SBT success rates

The SBT success rate in NAVA was high and comparable to PSV. All patients passed the SBT in PSV, which was unexpected, since most reports point to approximately 20% failure in the first SBT [2, 42, 43]. Possible explanations for such a high success rate could be that our study population was not too sick or that the ICU team’s decision to submit the patients to an SBT was delayed. However, our population had high SAPS 3 scores at admission, almost half had a history of chronic lung disease and, despite passing the SBTs in PSV, 25% of them required reintubation. We applied low values of inspiratory support and PEEP during the SBTs [1, 2, 8, 44] and ΔEAdi values during the trials were similar to values reported during mechanical ventilation weaning [21, 45]. However, we can’t rule out that this level of inspiratory support prevented clinicians from identifying patients not ready to breathe without assistance [46]. We believe that a reasonable explanation for the high success rate of SBTs in our population is that clinicians tend to perform badly when evaluating patients in SBTs and may have missed clinical signs of intolerance [47, 48].

Three patients failed the SBT in NAVA but, because our protocol mandated that clinicians used the result of the SBT in PSV, they were extubated. One of these patients needed to be reintubated. Given the preliminary nature of our trial, we were underpowered to detect differences in sensitivity and specificity to predict extubation failure between NAVA and PSV, and therefore we consider it premature to speculate if NAVA may improve the sensitivity and positive predictive value and reduce the specificity and negative predictive value of the SBT. The reintubation rate was relatively high, and could be a reflection of the severity of illness at ICU admission, measured by the SAPS3, and of the ICU profile, a unit that is specialized in care for patients with respiratory failure and need for mechanical ventilation in a large university hospital. Moreover, given the small number of participants, the confidence interval around the reintubation rate is large, and although Boles et all report an average 13% reintubation rate in 2007 [2], a large observational, multicentric study reported 29% of extubation failure [49].

Our study has several limitations. We recruited a small, convenience sample, because we were using NAVA during SBTs for the first time. This prevented the study from having enough power to detect differences in the success rate of SBTs in NAVA and PSV, and from comparing the predictive values for the two types of SBTs. The patients came from a single respiratory ICU that admits high-complexity patients, which might explain the high incidence of patient ventilator asynchrony on the day of extubation. The success of the SBT is a subjective outcome, dependent on observer experience, and may have been influenced by knowledge that participants were in a clinical trial. Finally, we used the EAdi, not electromyography, to identify patients’ inspiratory efforts; therefore, inspiratory efforts initiated by accessory muscles were not detected.

## Conclusions

We conclude that, during SBTs, NAVA reduces patient-ventilator asynchrony index compared to PSV while keeping a similar breathing pattern. We also conclude that SBTs in NAVA are safe, feasible, and have equivalent SBT success rates when compared to PSV. Patients who are considered ready for mechanical ventilation liberation and are being ventilated with NAVA may be submitted to an SBT in NAVA using the same objective criteria for SBT failure in PSV.

## Additional files

Additional file 1: Table S1. Patient’s ventilatory parameters at baseline. Ventilatory parameters for each study participant immediately before entering the trial. (DOCX 19 kb)

Additional file 2: Table S2. Individual patient’s ventilatory parameters during the SBTs and clinical outcome. Ventilatory parameters for each study participant during the SBTs, outcome of the each SBT (pass or fail) and outcome of the extubation (failure: yes of no). (DOCX 19 kb)

## Abbreviations

AI: Asynchrony index
CI: Confidence interval
COPD: Chronic obstructive pulmonary disease
CPAP: Continuous positive airway pressure
EAdi: Electrical activity of the diaphragm
ICU: Intensive care unit
IQR: Interquartile range
NAVA: Neurally adjusted ventilatory assist
Paw: Peak airway pressure
PEEP: Positive end-expiratory pressure
PSV: Pressure Support Ventilation
RR: Respiratory rate
SAPS 3: Simplified acute physiology score
SBT: Spontaneous breathing trials
TIneural: Neural inspiratory time
V_T_: Tidal volume
ΔEAdi: Delta electrical activity of the diaphragm: defined as EAdi peak minus EAdi minimal
