# Supplementary material for: Neurally Adjusted Ventilatory Assist (NAVA) or Pressure Support Ventilation (PSV) during spontaneous breathing trials in critically ill patients: a crossover trial
Source: BMC Pulm Med. 2017 Nov 7;17:139. doi: 10.1186/s12890-017-0484-5 (PMC5678780; doi:10.1186/s12890-017-0484-5)
Supplement: Supplementary file 1 — Patient’s ventilatory parameters at baseline. Ventilatory parameters for each study participant immediately before entering the trial. (DOCX 19 kb) [file 12890_2017_484_MOESM1_ESM.docx]

Table S1. Patient’s ventilatory parameters at baseline

| ID | V_T_/Kg (mL/Kg) | ΔEadi (µV) | RR (rpm) | PSV (cmH_2_O) | PEEP (cmH_2_O) | Cycling off | FIO_2_ |
| --- | --- | --- | --- | --- | --- | --- | --- |
| 1 | 6.1 | 2.4 | 16 | 10 | 5 | 30% | 0.3 |
| 2 | 5.5 | 32.0 | 15 | 17 | 7 | 35% | 0.4 |
| 3 | 6.8 | 14.4 | 17 | 12 | 6 | 40% | 0.35 |
| 4 | 5.9 | 6.2 | 33 | 12 | 5 | 35% | 0.3 |
| 5 | 5.8 | 7.8 | 31 | 7 | 8 | 20% | 0.35 |
| 6 | 7.2 | 1.5 | 18 | 10 | 8 | 30% | 0.3 |
| 7 | 8.4 | 3.9 | 22 | 9 | 8 | 30% | 0.4 |
| 8 | 4.6 | 5.8 | 31 | 8 | 6 | 40% | 0.35 |
| 9 | 5.4 | 6.3 | 23 | 6 | 6 | 30% | 0.3 |
| 10 | 7.2 | 6.2 | 14 | 7 | 6 | 25% | 0.25 |
| 11 | 6.0 | 10.4 | 34 | 8 | 8 | 30% | 0.35 |
| 12 | 4.7 | 5.5 | 33 | 10 | 10 | 15% | 0.3 |
| 13 | 6.5 | 1.8 | 18 | 10 | 6 | 30% | 0.3 |
| 14 | 10.9 | 6.8 | 22 | 10 | 8 | 30% | 0.35 |
| 15 | 7.2 | 20.4 | 12 | 8 | 5 | 60% | 0.35 |
| 16 | 7.4 | 6.9 | 20 | 6 | 8 | 25% | 0.35 |
| 17 | 5.0 | 4.2 | 38 | 10 | 5 | 25% | 0.3 |
| 18 | 7.5 | 30.5 | 15 | 12 | 6 | 40% | 0.5 |
| 19 | 6.4 | 7.3 | 18 | 15 | 8 | 50% | 0.5 |
| 20 | 7.2 | 22.2 | 24 | 10 | 5 | 30% | 0.35 |

Footnote: V_T_/kg: tidal volume per kilogram of predicted body weight; ΔEAdi: Delta Electrical activity of the diaphragm; RR: respiratory rate; PSV: Pressure Support Ventilation level on the day of the study; PEEP: positive end-expiratory pressure on the day of the study; FIO_2_: inspired fraction of oxygen. Values for V_T_/kg, ΔEAdi and RR are average values obtained from ventilator waveform recording and processing.
