# Supplementary material for: Neurally Adjusted Ventilatory Assist (NAVA) or Pressure Support Ventilation (PSV) during spontaneous breathing trials in critically ill patients: a crossover trial
Source: BMC Pulm Med. 2017 Nov 7;17:139. doi: 10.1186/s12890-017-0484-5 (PMC5678780; doi:10.1186/s12890-017-0484-5)
Supplement: Supplementary file 2 — Individual patient’s ventilatory parameters during the SBTs and clinical outcome. Ventilatory parameters for each study participant during the SBTs, outcome of the each SBT (pass or fail) and outcome of the extubation (failure: yes of no). (DOCX 19 kb) [file 12890_2017_484_MOESM2_ESM.docx]

Table S2. Individual patient’s ventilatory parameters during the SBTs and clinical outcome

| ID | ΔEAdi PSV | V_T_/Kg PSV | RR PSV | ΔEAdi NAVA | V_T_/Kg NAVA | RR NAVA | NAVA level | SBT in NAVA | Extubation failure |
| --- | --- | --- | --- | --- | --- | --- | --- | --- | --- |
| 1 | 7.7 | 5.6 | 17 | 7.1 | 7.5 | 15 | 0.8 | Pass | No |
| 2 | 20.6 | 4.8 | 15 | 29.4 | 5.5 | 14 | 0.2 | Pass | No |
| 3 | 21.3 | 6.0 | 20 | 18.2 | 7.0 | 18 | 0.4 | Pass | Yes |
| 4 | 7.7 | 4.5 | 39 | 4.1 | 4.1 | 34 | 1.4 | Pass | Yes |
| 5 | 8.2 | 5.0 | 31 | 5.2 | 6.2 | 28 | 1.2 | Fail | Yes |
| 6 | 3.5 | 6.1 | 26 | 4.4 | 6.8 | 22 | 1.4 | Pass | No |
| 7 | 7.7 | 6.2 | 30 | 10.3 | 6.7 | 35 | 0.7 | Pass | No |
| 8 | 5.6 | 4.8 | 27 | 8.4 | 6.5 | 27 | 1 | Pass | Yes |
| 9 | 6.4 | 4.8 | 26 | 8.3 | 4.9 | 28 | 0.9 | Fail | No |
| 10 | 5.8 | 6.2 | 16 | 5.0 | 5.7 | 17 | 0.7 | Pass | No |
| 11 | 10.2 | 6.1 | 24 | 12.9 | 6.3 | 32 | 0.5 | Pass | No |
| 12 | 10.6 | 4.6 | 31 | 7.5 | 4.8 | 24 | 0.8 | Pass | No |
| 13 | 5.2 | 5.1 | 21 | 7.8 | 5.3 | 23 | 0.8 | Pass | Yes |
| 14 | 8.2 | 5.4 | 30 | 12.3 | 6.1 | 30 | 0.7 | Pass | No |
| 15 | 13.1 | 6.8 | 11 | 27.2 | 6.3 | 12 | 0.2 | Pass | No |
| 16 | 24.3 | 10.1 | 22 | 4.8 | 5.6 | 37 | 0.8 | Fail | No |
| 17 | 18.9 | 5.2 | 28 | 11.5 | 6.3 | 29 | 1 | Pass | No |
| 18 | 33.5 | 5.6 | 15 | 37.1 | 5.6 | 17 | 0.1 | Pass | No |
| 19 | 11.7 | 5.5 | 20 | 22.9 | 8.4 | 17 | 0.4 | Pass | No |
| 20 | 22.9 | 6.1 | 26 | 40.6 | 5.6 | 30 | 0.2 | Pass | No |

Footnote: PSV: pressure support ventilation; NAVA: Neurally adjusted ventilatory assist; ΔEAdi: Delta Electrical activity of the diaphragm, in µV; V_T_/kg: tidal volume per kilogram of predicted body weight, in mL/Kg; RR: respiratory rate; NAVA level, in µV/cmH_2_O; SBT in NAVA: outcome of the SBT in NAVA; Extubation failure: need for reintubation within 48hs after extubation. Values for V_T_/kg, ΔEAdi and RR in NAVA and PSV are average values were obtained from ventilator waveform recording and processing.
